# Supplementary material for: Examining Obedience Training as a Physical Activity Intervention for Dog Owners: Findings from the Stealth Pet Obedience Training (SPOT) Pilot Study
Source: Int J Environ Res Public Health. 2021 Jan 21;18(3):902. doi: 10.3390/ijerph18030902 (PMC7908228; doi:10.3390/ijerph18030902)

Supplementary Materials for "Examining Obedience Training as a Physical Activity Intervention for Dog Owners: Findings from the Stealth Pet Obedience Training (SPOT) Pilot Study"

Appendix: Further details on the statistical analysis.

The objective of this pilot study was to determine whether a 6-week dog obedience training class improves physical activity (PA) among inactive dog owners. The primary outcomes were changes from baseline in average steps/day and in daily minutes of MVPA (defined as intensity ≥3 metabolic equivalents) at 6 and 12 weeks. Secondary outcomes included dog walking days per week, daily minutes of sedentary behavior, strength of the dog-owner bond (emotional closeness with dog), dog walking self-efficacy, and social support (from the dog) for walking.

To accomplish this objective, we analyzed data from the SPOT Study (NCT: 04329741), which randomized 41 participants to the a 6-week dog obedience training class or a waitlist control. Eligibility criteria and study procedures are described in the main manuscript. Here, we provide additional details on the statistical approach to assess the intervention on the primary and secondary outcomes.

We sought to learn the difference in the average outcome if all participants had obedience training versus if the same participants over the same time period had not. Our target causal parameter was, thus, the sample effect for the n participants:

$$\frac{1}{n}\sum_{i=1}^{n} Y_{i,t}(1) - Y_{i,t}(0)$$

where $Y_{i,t}(1)$ represents the counterfactual outcome at time t for participant i under the intervention and $Y_{i,t}(0)$ denotes the corresponding counterfactual outcome under the control. Since SPOT was a randomized trial, the sample effect can be identified from the observed data distribution. Specifically, to estimate the intervention effect, we could simply take the difference in the average outcomes between groups. However, accounting for baseline covariates during the analysis can improve precision and thereby power in randomized trials.

Therefore, we estimated the intervention effect with targeted maximum likelihood estimation (TMLE), which provides efficiency gains over an unadjusted approach (e.g. the Student's t-test) by adjusting for chance imbalance between randomized arms on baseline predictors of the outcome [1-2]. Briefly, TMLE combines estimates of the "outcome regression" (e.g. expected change in physical activity, given the treatment arm and adjustment set) with estimates of the "propensity score" (e.g. conditional

probability of being assigned to the intervention group, given the adjustment set). As a result, TMLE achieves a number of desirable properties, such as double robustness, precision, and valid statistical inference even when using data-adaptive algorithms.

Given the limited number of randomized participants (*N=41*), we used Adaptive Pre-specification to optimally adjust for covariate imbalance between randomized arms, while preserving Type-I error control [3]. The candidate adjustment variables, assessed at baseline, were sex, BMI, emotional closeness with dog, dog walking self-efficacy, social support from dog for walking, and satisfaction with dog's behavior. At most, we adjusted for one variable in the outcome regression and one variable in the propensity score. Leave-one-out cross-validation was used to select the candidate TMLE that minimized the estimated variance. In sensitivity analyses, we implemented the unadjusted estimator as the difference of the arm-specific mean outcomes.

All physical activity outcomes were parameterized in terms of the change from baseline. A sample size of 40 adults was selected to provide at least 80% power to detect differences between randomized groups of at least 1,500 average steps/day and at least 76 minutes/week of MVPA. Such differences are consistent with meeting 50% of the aerobic physical activity guidelines, which have been associated with improvements in cardiorespiratory fitness among previously sedentary individuals [4-5].

Statistical inference was based on the estimated influence curve and the Student's *t*-distribution to account for limited sample sizes. Hypothesis testing was conducted with a two-sided test at the 5% significance level. Primary analyses excluded participants whose outcome assessments were missing at the timepoints of interest; sensitivity analyses adjusted for potentially differential measurement.

References

1. van der Laan, M. J.; Rose, S. *Targeted Learning: Causal Inference for Observational and Experimental Data*; Springer Series in Statistics; Springer-Verlag: New York, 2011.
2. Rosenblum, M.; van der Laan, M. J. Simple, efficient estimators of treatment effects in randomized trials using generalized linear models to leverage baseline variables. *Int. J. Biostat.* **2010**, *6*.
3. Balzer, L. B.; van der Laan, M. J.; Petersen, M. L.; SEARCH Collaboration. Adaptive pre-specification in randomized trials with and without pair-matching. *Stat. Med.* **2016**, *35*, 4528–4545.
4. Church, T. S.; Earnest, C. P.; Skinner, J. S.; Blair, S. N. Effects of different doses of physical activity on cardiorespiratory fitness among sedentary, overweight or obese postmenopausal women with elevated blood pressure: A randomized controlled trial. *JAMA* **2007**, *297*, 2081–2091.

5. Jordan, A. N.; Jurca, G. M.; Locke, C. T.; Church, T. S.; Blair, S. N. Pedometer indices for weekly physical activity recommendations in postmenopausal women. *Med. Sci. Sports Exerc.* **2005**, *37*, 1627–1632.

Supplementary Figure S1. Study CONSORT diagram

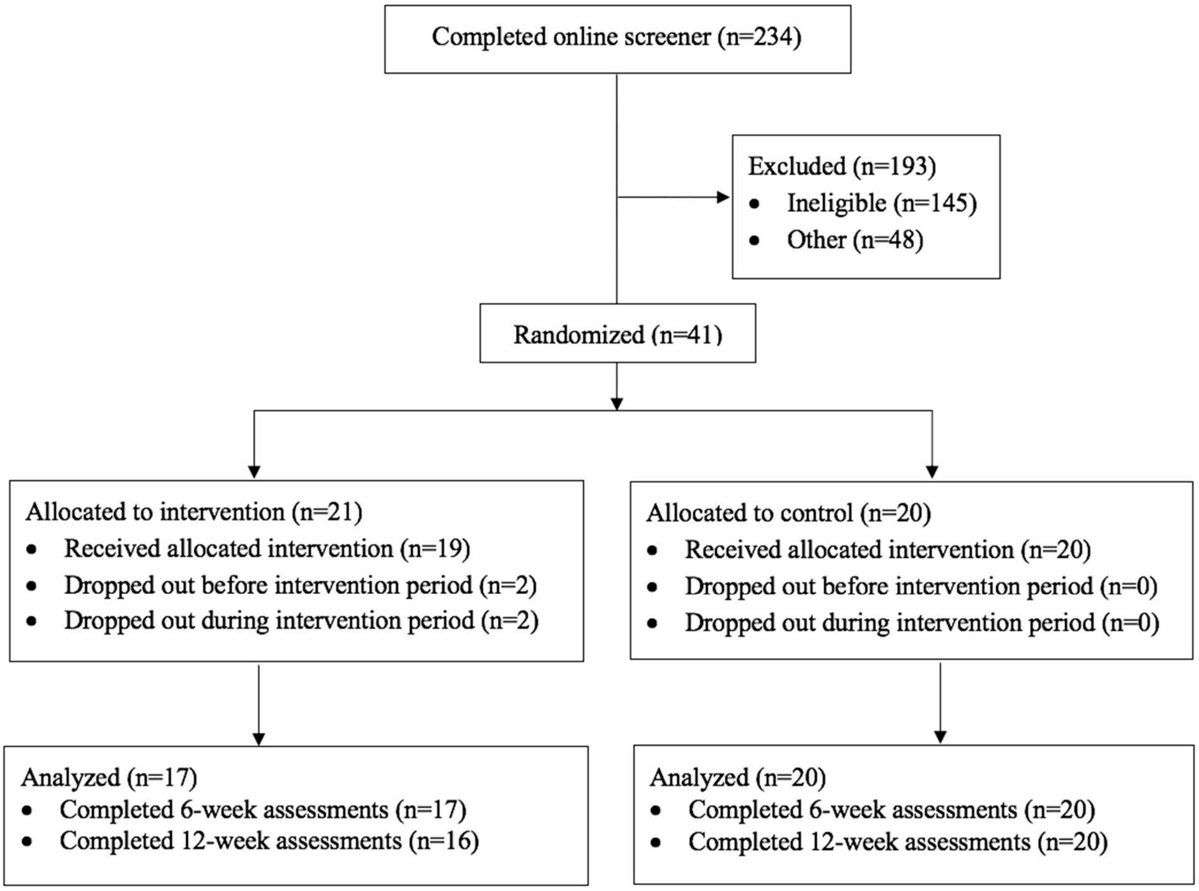

Supplement: Supplementary file 1 [file ijerph-18-00902-s001.pdf]
